# Effectiveness of community health workers on identification and mobilization of persons living with epilepsy in rural Rwanda using a validated screening tool

**DOI:** 10.1186/s12960-022-00704-5

**Published:** 2022-01-21

**Authors:** Fidele Sebera, Peter Dedeken, Jeannine Kayirangwa, Josiane Umwiringirwa, Delphine Kajeneza, Nicole Alves dos Reis, Tim Leers, Dirk E. Teuwen, Paul A. J. M. Boon

**Affiliations:** 1 Department of Neurology, Brothers of Charity, CARAES Neuro-psychiatric Hospital, Ndera, Kigali, Rwanda; 2 Centre Hospitalier Universitaire Kigali, Kigali, Rwanda; 3 Department of Neurology, Ghent University Hospital, Ghent, Belgium; 4 Heilig Hart Ziekenhuis, Lier, Belgium; 5 UCB Pharma, Brussels, Belgium; 6 4Brain, Ghent, Belgium; 7 Department of Mental Health and Neurology, Ruhengeri Referral Hospital, Musanze, Rwanda; 8 Department of Neurology, National University Hospital FANN, University of Cheikh Anta Diop, Dakar, Senegal; 9 Dataroots bvba, Leuven, Belgium

**Keywords:** Community health workers, Work engagement, Epilepsy, Health surveys, Rwanda

## Abstract

**Introduction:**

Engagement and training of community health workers (CHWs) have demonstrated their value in different conditions. Despite repeat epilepsy trainings of CHWs in Northern Rwanda, the treatment gap remained high. We hypothesized that effectiveness of CHWs on mobilization of patients living with epilepsy (PwE) could be improved using a validated tool for epilepsy screening.

**Methods:**

CHWs associated with health centers (HCs) of Gataraga, Kimonyi and Karwasa attended a 1-day training on epilepsy and Limoges epilepsy screening questionnaire (Kinyarwanda version). Thereafter, CHWs screened households in their villages for persons with one or more positive answer. CHWs then accompanied positively screened persons to a consultation for clinical evaluation and diagnosis by neurologists, and demographic data were collected. CHW variables were collected retrospectively.

**Results:**

A total of 1308 persons were screened positive by 281 CHWs. Clinical diagnosis of epilepsy was confirmed in 589 and in 93 additional unscreened PwE, presenting voluntarily at the consultation. Pre-intervention number of 48 PwE increased to 682 after, a 14.2-fold increase. The overall treatment gap amounted to 93.0%. The age distribution of male PwE preponderance at younger age inverted to females at older age.

CHW characteristics showed non-significant differences within and across HCs. Logistic regression did not relate CHW age, gender, and experience to screening results.

**Discussion:**

Equipping CHWs with a validated screening tool was effective in identifying and mobilizing PwE in a short time frame and offers opportunity for future scaling. Nonetheless, barriers to sustainability of care will need to be addressed before.

**Supplementary Information:**

The online version contains supplementary material available at 10.1186/s12960-022-00704-5.

## Introduction

There are 70 million people living with epilepsy worldwide [1]. In sub-Saharan Africa, its prevalence varies widely between regions, with a consistent trend of higher prevalence in rural compared to urban settings [2]. In 2005, the national epilepsy prevalence in Rwanda was estimated at 49.3 per thousand with higher prevalence in rural areas. In 2017, a prevalence of 47.7 per thousand was observed in rural villages in the Northern province [3, 4]. The treatment gap in this region amounted to 91.5%, applying the ILAE treatment gap definition encompassing diagnostic and therapeutic deficits [5, 6]. Epilepsy diagnosis is made clinically based on the description of recurrent episodes, preferably confirmed by an experienced physician, eventually supported by diagnostic investigations, such as imaging or EEG [3]. Leading causes of the epilepsy treatment gap include limited access to treatment, lack of primary trained health workers, social stigma, misinformation, and traditional beliefs [6].

Different measures to close the treatment gap have been proposed and the World Health Organization mental health Gap Action Plan (mhGAP) outlined a guide to implementation [6–8]. Accordingly, Rwanda included epilepsy in the activity package of primary healthcare professionals, improved the access to and the availability of anti-seizure medication (ASM) in primary health centers (HCs) and expanded diagnostic EEG availability. The Rwandan Organization against Epilepsy (ROAE) drove capacity building through training of mental health physicians, nurses and psychologists in primary HCs and district hospitals as epilepsy diagnosis and management relies on their expertise [3]. A community-based healthcare insurance was developed to cover up to 90% of medical costs, if a subscription fee is paid [9].

Another measure is the engagement of community leaders (traditional healers and religious leaders) and community health workers (CHWs) [6]. Indeed, CHWs are key members of rural communities and act as a link between patients and existing healthcare structures through mobilization of patients and education on health-related topics. They have previously illustrated their contribution in maternal health and communicable diseases [10, 11]. In Rwanda, CHW engagement in the Rapid SMS project for mothers and childcare, decreased maternal mortality by 78% between 2005 and 2015 [10, 12].

Few projects in Rwanda involved CHWs to narrow the epilepsy treatment gap. In one vertically integrated epilepsy care program, 4429 CHWs of 85 HCs in four health districts were trained to change attitudes of the community towards PwE through education and to engage them in psychoeducational groups [11]. In parallel to this project, the ROAE provided an initial and repeat epilepsy training to more than 400 CHWs in the Northern Province in 2014 and 2017, assuming training alone would contribute to treatment gap narrowing. However, in 2017, the gap remained significant in several HCs within these regions at 91.5% [4].

Effectiveness of CHW engagement is influenced by different factors including availability of appropriate tools, correct incentives, appropriate referral systems, and recognition [13]. We hypothesized that improved engagement of CHWs for identification and referral of PwE could be achieved by equipping them with a validated tool for epilepsy screening, improving guidance for referral to HCs and ensuring capacity for diagnosis and follow-up. The Limoges questionnaire has been validated as an epilepsy screening tool in Mauritania with sensitivity and specificity of 95.1% and 65.6%, respectively [14]. It has demonstrated its value in multiple countries throughout sub-Saharan Africa [15].

We report the effectiveness of CHW engagement on screening and mobilization of PwE after implementation of these measures.

## Materials and methods

### Study rationale

Within the framework of an interventional study on epilepsy and depression as co-morbidity, PwE were to be recruited from different villages covered by the HCs of Karwasa, Gataraga and Kimonyi in the Musanze district in Northern Rwanda. These HCs in a rural setting were selected allowing for varying geographical sectors, distance to the district hospital and different center specifications, upon discussion with the district hospital staff and after a study feasibility visit. Qualified mental health staff with experience in first-line epilepsy care was present in all centers.

Based on the nationwide epilepsy prevalence data of 49.3 per thousand and given an approximate population of 72,635 villagers, we estimated a prevalence of more than 3500 PwE in this region [16]. However, during a baseline period of 3 months between March and June 2018, only 48 possible study participants were identified at these HCs and we assumed an important treatment gap, despite previous CHW training on epilepsy in this area in 2014 and 2017. Baseline patient numbers were 18, 11 and 19 at the Gataraga, Kimonyi and Karwasa HC, respectively.

### Ethical considerations

The study was approved by the College of Medicine and Health Sciences—Institutional Review Board (Kigali, Rwanda) [461/CMBS-IRB/2016]. Verbal consent was approved considering possible illiteracy and the culturally related skepticism to the officialdom of a written consent if applied to conduct a screening survey.

As it was considered unethical to provide diagnosis without putting measures in place for long term treatment and follow-up, a situational analysis was conducted on the base case using the hypothesized treatment gap [17, 18]. It was a concerted effort of the study team, CHW supervisors, HC and district hospital staff and the pharmacy. As a result, measures to ensure access to ASMs for newly diagnosed PwE were implemented. Additional healthcare professionals were recruited in the respective HCs. In each village, psycho-educational groups were created bringing together CHWs and PwE, aiming to increase epilepsy knowledge and experience sharing, reduce stigma and improve income generation for PwE. These were organized monthly and chaired by a contracted social worker.

### Screening tool

The Limoges epilepsy screening questionnaire has previously been translated into Kinyarwanda and adapted to the sociocultural context of Rwanda [3]. It consists of five epilepsy related questions which require a dichotomous ‘yes/no’ answer (see Additional file 1). If a single question is answered ‘yes’, the respondent is considered positively screened and referred for confirmation of a diagnosis of epilepsy to an experienced healthcare professional.

### Recruitment and training of CHWs

In June 2018, CHWs of all villages covered by the Karwasa, Gataraga and Kimonyi HCs, were invited to attend a 1-day training session. In addition, at the recommendation of the supervisor at the district hospital, CHWs of selected neighboring villages of the adjacent villages were also invited. Preparatory meetings with the district hospital staff, executive secretaries and nurses supervising the CHWs, preceded the trainings.

In contrast to previous more general epilepsy awareness trainings, CHWs were trained by a neurologist and a project coordinator, specifically on signs and symptoms of epilepsy, which are included in the epilepsy screening questionnaire. During training of the screening questionnaire, questions were pre-tested with CHWs to ensure clarity and understanding. A role-play was organized on administering all five questions to household members.

CHWs received the recommended per diem, as per the Official Gazette, for their attendance at the training as well as for the working days in their villages completing household surveys [19].

### Structured screening methodology

CHWs were instructed to ask each person all five questions and record the answer on a paper version of the questionnaire. They were asked to also include those who had a previous diagnosis of epilepsy as PwE may had discontinued treatment or follow-up whilst having active seizures. For minors, an adult member of the household answered the questions.

For 1 day in June 2018, they performed household visits in their community according to a schedule agreed with the social worker.

To ensure maximal coverage of each village, the social worker coordinated a structured screening approach by assigning each CHW to a single part within the village. CHWs were instructed to visit every household in the assigned part. In contrast to a stringent door-to-door approach, they interviewed only those household members found at home at the time of screening. Return visits were not requested and no paper questionnaires were left for those missing from the family. To limit the administrative burden, CHWs were not requested to note how many persons were screened.

### Referral and epilepsy diagnosis of persons with positive screening

Any person who screened positive, was invited to a consultation with a neurologist or a resident-neurology at the nearest HC to confirm diagnosis.

CHWs and the social worker scheduled the consultations together. CHWs accompanied any positively screened at various dates in July 2018. When epilepsy diagnosis was confirmed, demographic data and epilepsy characteristics were recorded. PwE were invited to participate in psycho-educational–economic groups.

If villagers attended the consultation without having been interviewed by a CHW, they were equally seen by the physician, but their demographic data were excluded from demographic analyses.

### Data collection and statistical analysis

Screening results on paper, were transferred by single data entry into Excel and matched with the data recorded during the consultation. CHW characteristics (gender, age, and duration of CHW employment) were collected retrospectively. Descriptive analysis was performed in Excel. Chi-square test, fisher exact test and one-way ANOVA were applied to test for differences in means and for group differences. The relationship between CHW characteristics and survey results were analyzed with a logistic regression model, estimated using maximum-likelihood modelling and conducted in R package. *P* value < 0.05 was used as level of significance. Missing data were not imputed.

## Results

### CHW characteristics and training

A total of 319 CHWs from 114 villages attended the epilepsy training in groups of 50–60 CHWs over a 5-day period. CHWs from outside the catchment area of the respective HCs accounted for 12% (38/319). Despite a larger number of villages covered by the Karwasa HC, the number of trained CHWs per village was similar across health centers (see Table 1).

**Table 1:** Health Center specifications and CHW characteristics by center and by gender; chi-square test, *the Gataraga health center is equipped with a hospitalisation ward

Health center	Karwasa	Kimonyi	Gataraga*	Total
Cells covered [*n* (%)]	9 (53.0)	4 (23.5)	4 (23.5)	17 (100)
Villages covered [*n* (%)]	61 (60.4)	20 (19.8)	20 (19.8)	101 (100)
CHWs deployed/trained [*n* (%)]	170 (60.5)	56 (19.9)	55 (19.6)	281 (100)
Number of CHWs per village	2.77	2.8	2.75	2.78

Gender	Female	Male	Female	Male	Female	Male	Female	Male
*n* (%)	107 (64.1)	60 (35.9)	39 (69.9)	17 (30.4)	36 (65.5)	19 (34.5)	182 (65.5)	96 (34.5)
	*p* = 0.83							

Age	Female	Male	Female	Male	Female	Male	Female	Male
*n* (%)	50 (62.5)	30 (37.5)	23 (67.6)	11 (32.4)	13 (65.0)	7 (35.0)	86 (61.9)	48 (35.8)
Mean (year)	51.5	51.6	44.0	47.1	46,2	39.1	48.7	48.8
Grouped by age [*n* (%)]								
≤ 39 years	5 (10.0)	3 (10.0)	7 (30.4)	2 (18.2)	1 (7.7)	5 (71.4)	13 (15.1)	10 (20.8)
40–49 years	17 (34.0)	11 (36.7)	11 (47.8)	6 (54.5)	8 (61.5)	1 (14.3)	36 (41.9)	19 (39.6)
50–59 years	18 (36.0)	9 (30.0)	4 (17.4	1 (9.1)	4 (30.8)	0 (0.0)	26 (30.2)	10 (20.8)
≥ 60 years	10 (20.0)	6 (20.0)	1 (4.3)	2 (18.2)	0 (0.0)	1 (14.3)	11 (12.8)	9 (18.8)
	*p* = 0.99		*p* = 0.56		*p* = 0.03		*p* = 0.49	

Duration of experience	Female	Male	Female	Male	Female	Male	Female	Male
*n* (%)	54 (62.8)	32 (37.2)	23 (67.6)	11 (32.4)	13 (68.4)	6 (31.6)	90 (64.7)	49 (35.3)
Mean (year)	9.8	9.6	9.3	9.6	8.6	8.2	9.5	9.2
Grouped by experience [*n* (%)]								
≤ 4 years	4 (7.4)	4 (12.5)	4 (17.4)	3 (27.3)	1 (7.7)	0 (0.0)	9 (10.0)	7 (14.3)
5–9 years	3 (5.6)	5 (15.6)	5 (21.7)	3 (27.3)	4 (30.8)	3 (50.0)	12 (13.3)	11 (22.4)
≥ 10 years	47 (87.0)	23 (71.9)	14 (60.9)	5 (45.5)	8 (61.5)	3 (50.0)	69 (76.7)	31 (63.3)
	*p* = 0.17		*p* = 0.69		*p* = 0.74		*p* = 0.22	

CHW characteristics were available for nearly 50% engaged CHWs. Up to two in three were female. This gender preponderance did not affect the mean age within HCs, whereas an analysis by age groups differed significantly for the Gataraga HC, with younger male CHWs. Most CHWs had a longstanding experience, without a gender difference across HCs.

### Screening by CHWs

A total of 281 CHWs completed screening interviews in 101 villages and 1308 persons screened positive to at least one question (see Table 2). At Karwasa, having the largest catchment area, a greater number of persons screened positive compared to other centers. A mean and median of 4.7 and 4 (range 1–27) positively screened persons was referred per CHW, respectively.

**Table 2:** Screening, demographics, and baseline characteristics of PwE by center and by gender

Health center	Karwasa		Kimonyi		Gataraga		Total	
Screening & diagnosis								
Positive screening result	660		369		279		1308	
Epilepsy not confirmed [*n* (%)]	361 (54.7)		210 (56.9)		148 (53.0)		719 (55.0)	
Confirmed epilepsy [*n* (%)]	299 (45.3)		159 (43.1)		131 (47.0)		589 (45.0)	
	*p* = 0.61							
Gender	Female	Male	Female	Male	Female	Male	Female	Male
*n* [%]	152 (50.9)	147 (49.1)	118 (74.2)	41 (25.8)	77 (58.8)	54 (41.2)	347 (58.9)	242 (41.1)
	*p* ≤ 0.0001							

Demographics and baseline characteristics of persons confirmed with epilepsy								
Age	*n* = 291		*n* = 159		*n* = 130		*n* = 580	
Median	18.0		35.0		27.0		24.0	
Mean ± SD	23.0 ± 17.4		35.1 ± 21.4		30.4 ± 20.3		27.9 ± 19.9	
	*p* < 0.0001*							
Age by gender	Female	Male	Female	Male	Female	Male	Female	Male
*n* [%]	148 (50.9)	143 (49.1)	118 (74.2)	41 (25.8)	76 (58.5)	54 (41.5)	342 (59.0)	238 (41.0)
Median	23.5	14.0	40.0	15.0	32.0	20.5	31.0	15.0
Mean ± SD	26.4 ± 17.7	19.4 ± 17.7	39.9 ± 20.6	21.4 ± 17.4	33.8 ± 19.4	21.1 ± 20.9	25.5 ± 20.0	21.1 ± 17.7
	*p* = 0.0008*		*p* < 0.0001*		*p* = 0.0005*		*p* = 0.0065*	
Age category by gender [*n* (%)]	Female	Male	Female	Male	Female	Male	Female	Male
0–19 years	63 (42.6)	91 (63.6)	27 (22.9)	25 (61.0)	24 (31.6)	27 (50.0)	114 (33.3)	143 (60.1)
20– 39 years	48 (32.4)	35 (24.5)	31 (26.3)	9 (22.0)	21 (27.6)	15 (27.8)	100 (29.2)	59 (24.8)
≥ 40 years	37 (25.0)	17 (11.9)	60 (50.8)	7 (17.1)	31 (40.8)	12 (22.2)	128 (37.4)	36 (15.1)
	*p* = 0.0007		*p* < 0.0001		*p* = 0.049		*p* < 0.0001	
Income category [*n* (%)]	Female	Male	Female	Male	Female	Male	Female	Male
Number	149	147	117	39	74	53	340	239
Level 1	32 (21.5)	31 (21.1)	38 (32.5)	9 (23.1)	14 (18.9)	12 (22.6)	84 (24.7)	52 (21.8)
Level 2	72 (48.3)	66 (44.9)	67 (57.3)	26 (66.7)	43 (58.1)	21 (39.6)	182 (53.5)	113 (47.3)
Level 3	45 (30.2)	50 (34.0)	12 (10.3	4 (10.3)	17 (23.0)	20 (37.7)	74 (21.8)	74 (31.0)
	*p* = 0.77		*p* = 0.52		*p* = 0.1		*p* = 0.044	
Social security coverage [*n* (%)]	Female	Male	Female	Male	Female	Male	Female	Male
Number	150	145	117	40	75	53	342	238
No	99 (66.0)	87 (60.0)	52 (44.4)	26 (65.0)	43 (57.3)	29 (54.7)	194 (56.7)	142 (59.7)
Yes	51 (34.0)	58 (40.0)	65 (55.6)	14 (35.0)	32 (42.7)	24 (45.3)	148 (43.3)	96 (40.3)
	*p* = 0.29		*p* = 0.025		*p* = 0.77		*p* = 0.48	
	*p* = 0.021							

Chi-square test for differences, * using one-way ANOVA

All 1308 positive screens accepted to attend the neurology consultation.

**Fig. 1:**
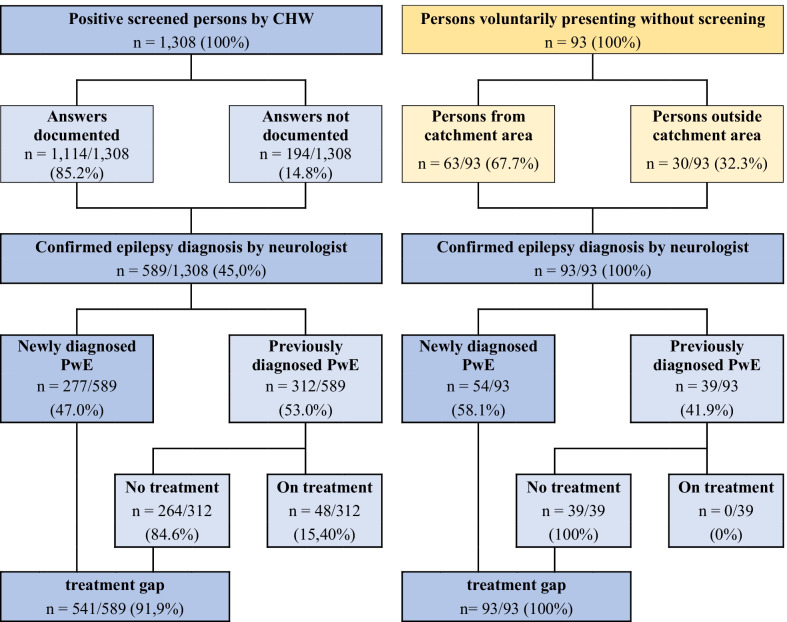
Left: distribution of patients who screened positive, confirmed epilepsy diagnosis and treatment gap. Right: distribution of persons voluntarily presenting without screening

Clinical diagnosis was confirmed in 589 (45%) (see Fig. 1). On average, 2.1 PwE were referred per CHW (589PwE/281CHWs). In total 277 (47%, 277/589) new cases were identified. The ratio of newly to previously diagnosed PwE in Karwasa, Gataraga and Kimonyi was 42%; 58% and 47%, respectively. Of the 312 patients with a previous diagnosis of epilepsy, only 48 were taking ASM, resulting in a treatment gap of 91.9% (*n* = 541/589) in the screened population. The treatment gap was lower in Gataraga at 86.2% versus Karwasa and Kimonyi with 92.2 and 92.5%, respectively.

In addition, 93 persons, who had not been attended by a CHW, presented voluntarily and unexpectedly to the neurology consultation (see Fig. 1). Of those, 63 (67.7%) came from within the catchment area. All were clinically confirmed cases and 41.9% had been previously diagnosed with epilepsy, yet none were on ASM. The treatment gap including these cases amounted to 93.0%.

**Fig. 2:**
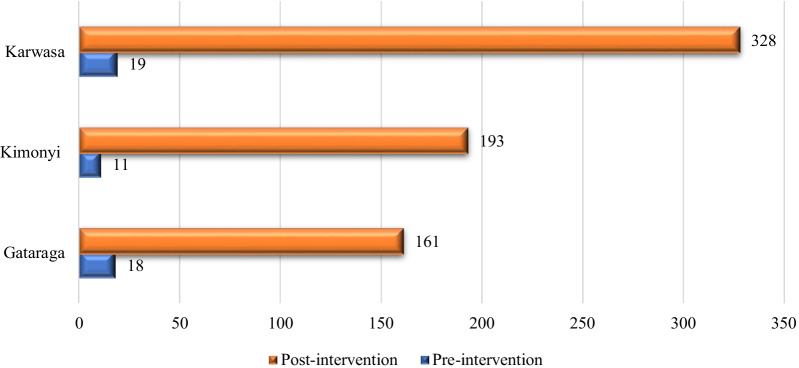
Number of PwE per HC pre- and post-intervention

Before the intervention, only 48 PwE were attending the three HCs. Post-intervention, we observed a 14.2-fold increase in number of PwE across these sectors. The increases were 17.3, 8.9 and 17.5-fold at the HCs of the Karwasa, Gataraga and Kimonyi HCs, respectively, not significantly differing between sites (*p* = 0.063) (see Fig. 2) With 82.6% of CHWs bringing forward at least one positively screened with confirmed epilepsy and 85% of them referring a person without epilepsy diagnosis, no clear screening bias was noted.

We performed a logistic regression to assess whether CHW age, gender, and experience, influenced the screening results, including CHWs who had referred at least one confirmed PwE. We hypothesized that gender, age, and experience would increase the likelihood of a positive screen to be confirmed with epilepsy. Higher age and longer experience had a small effect, which did not reach significance (*p* > 0.05).

### Baseline demographics of PwE

Baseline demographics of confirmed PwE are summarized in Table 2. Significant differences between centers were observed for gender with more female patients in Kimonye and Gataraga compared to Karwasa, with respective ratios female/male of 2.32, 1.37 and 0.97. Mean age of PwE was also lower in Karwasa.

Interestingly, the distribution by age groups showed a reversal from a male preponderance in the younger age group to a female preponderance in the older age group, all significant within each center. Significant differences in mean between sexes with female PwE having higher age confirmed these observations (see Table 2).

Using logistic regression, we assessed whether CHW characteristics could explain these differences. We hypothesized that age, gender, and experience of CHWs predicted gender preference, yet no statistically significant relation was observed.

The income level, labelled according to the Rwandan standards, demonstrated no gender difference within and across HCs. Data on social security coverage was only available for 42.0% of PwE, and was not influenced by gender, except for the site of Kimonye with more males not having a health insurance coverage (see Table 2).

## Discussion

### CHW engagement and effectiveness

The pivotal role of CHWs has already been recognized in communicable diseases, e.g., HIV, Ebola, malaria, tuberculosis, among others, as well as non-communicable diseases, e.g., hypertension, diabetes, among others, yet only infrequently have they been engaged in epilepsy [11, 20–23].

In Mozambique, inclusion of 1161 CHWs in the implementation of epilepsy in primary care, resulted both in increased mobilization and long-term follow-up of PwE, reducing the treatment gap by 3% and increasing the number of newly diagnosed PwE twofold after 3 years [24]. In Rwanda, 4429 CHWs of four districts were trained on epilepsy and were engaged in psycho-educational groups for PwE, which resulted after 4 years in the identification of 6330 PwE of whom more than a 30% were on treatment after 2 years [11].

In contrast to these long-term programs, we opted for a one-off, short-term coordinated action with a single day training and a short screening period, quickly followed by referral for confirmation of diagnosis. We confirmed our hypothesis of an existing treatment gap as previously documented in Rwanda [3, 25]. A total 541 PwE was referred for epilepsy follow-up and treatment, which is a decrease of more than 15% of the initially assumed diagnosis gap, which still remains large [4]. Repeat screening may, therefore, be needed, yet the optimal interval is unclear.

Of interest, we observed a higher diagnostic gap and a lower therapeutic gap in Gataraga, the most remote center from the district hospital. Whereas a higher number of newly diagnosed patients can be explained by a sector based underdiagnosis, the lower therapeutic gap in Gataraga may be explained by its geographical properties or the health services provided as it hosts a hospitalization ward. If future studies demonstrate that specific healthcare services impact the treatment gap, this may be considered as a strategy in future health programs.

Whereas other CHW training initiatives focused only on epilepsy knowledge and awareness, we combined general epilepsy training with training of specific signs and symptoms of epilepsy, aligned to the questions of a validated screening tool [14, 15]. This different methodology may explain the higher number of confirmed cases per trained CHW (2.1 after a single day; 589 PwE/280 CHWs) compared to an earlier long term project (1.5 over 4 years; 6330 PwE/4429 CHWs) [11].

CHWs demonstrated high engagement as they ensured that positively screened persons respected their scheduled appointment with the neurology team. The confirmation of a clinical diagnosis of epilepsy by a neurologist was deemed necessary to decrease any misdiagnosis. Although the presence of a neurologist at the consultation, compared to a mental health nurse in daily practice at the HC, may have increased the willingness to attend the consultation, we consider it unlikely to have influenced screening results.

On the other hand, we observed a high variability in the number of patients referred to the HC from 1 to 27 persons per CHW, possibly reflecting different levels of engagement. Possible explanations include epilepsy training effect and interpretation, conflicting tasks, bias in administration of the tool or motivational aspects. Future studies are needed to evaluate the drivers for this variability using the CHW individual questionnaire [26].

In our project, CHWs were not instructed to conduct a solid door-to-door approach and may have missed household members when executing screening during daytime, possibly resulting in a selection bias. More females, involved in the household, and children not attending school, may have been screened and detected. This may have been the case for the HC of Kimonye, with a female/male ratio of newly diagnosed PwE of 2.32.

Interestingly, we observed a spill-over effect of the project within and outside the catchment area since non-screened persons presented unexpectedly to the neurology consultation. The confirmation of epilepsy in all those subjects may indicate that epilepsy as a disease may be recognized yet does not necessarily lead to care seeking.

### CHW use of tools

In Belize, CHWs reported the need for appropriate tools and equipment, such as a blood pressure monitor [27]. The use of tools has also been recommended to improve CHW effectiveness [13]. The value of validated questionnaires by CHWs has been demonstrated in South Africa during screening for cardiovascular diseases [28, 29]. A cell-phone based screening tool for hearing impairment deployed to CHWs equally has proven to be accurate for screening [30, 31].

In resource limited settings, epilepsy is a clinical diagnosis. Following our results, we advocate the use of the validated screening questionnaire as the optimal tool for mobilization of PwE. It is, however, not suited for follow-up of patients. The positive predictive value of screening tool was 45%. This yield needs further analysis as future screening or mobilization projects may want to increase both human resource allocation and return on screening.

We did not find CHW gender, age, or duration of experience to relate significantly to screening results. We found no difference in number of CHWs referring positively screened with and without confirmed diagnosis. In addition, the observed inversion of gender preponderance with male predominance in younger age group to a female preponderance in the older age groups was not significantly influenced by CHW characteristics. This age shift has been observed in Zambia as well, which may be explained by a survival bias, age-specific etiologies, care seeking patterns, gender-dependent risk factors, or competing mortality risks [4, 32].

### Considerations on sustainability, scalability, and future directions

Sustainability of epilepsy care was assessed before the project start and the situational analysis identified ASM availability, human resource capacity in HCs and possible financial restraints for diagnosed PwE as gaps to ensuring long term care [17, 18]. Up to 66% of PwE were in the lower two-tier income levels and 6 in 10 PwE had no access to social security. This may explain the low adherence to treatment in previously diagnosed PwE. Financial concerns may delay care seeking and directly contribute to the diagnosis gap. To address this barrier, financial support was addressed by the organization individualized psycho-economic activities through micro-financing credits and the creation of psycho-educational groups which involved the CHWs. CHWs thus proved instrumental both in screening and ensuring sustainability of care.

CHWs were trained on the disease and the tool for 1 day, which may prove enough for one-off actions. When a tool would permanently be rolled out to CHWs with epilepsy being part of their work package, the need for repeated training will require further investigation. Single epilepsy trainings have demonstrated long term effects on knowledge, attitudes and practices in CHWs [33]. Yet, appropriate use of tools may require repeat trainings.

Another challenge was the anticipated workload for the CHWs. Both single day training and screening was thought not to interfere significantly with ongoing daily tasks at hand. The project, however, resulted in additional workload as the CHW accompanied referred persons to the neurology consultation and given their involvement in psycho-educational groups afterwards. Although mental health is currently included in the task description of Rwandan CHWs, scaling up to large screening projects will add workload, which may require revision of work packages or increased CHW capacity. Future projects may also address the question whether CHWs could participate in distribution of ASMs, which has shown to decrease workload for HC staff engaged in treatment of HIV patients [20]. In addition, measures to prevent loss of data must be put in place, as we observed up to 15% missing questionnaires. A digital version of the screening tool for iOS and Android is currently under development.

Our approach with trained CHWs, equipped with a specific screening tool, seemed to be at least as effective in terms of number of patients mobilized compared to longer term programs and may offer an opportunity for future projects [34, 35]. However, the barriers to sustainability of care will need to be addressed before future scaling.

## Limitations

The interpretation of our data relative to prevalence calculations is limited as neither prevalence nor incidence can be derived. CHWs were not asked to apply a stringent door-to-door methodology and did not record the number of persons and households interviewed.

Second, CHWs may not have visited all households in the village or have screened all members of a household, resulting in missed households/household members because of agricultural activities or school attendance. From a scalability perspective on mobilization campaigns, we feel that well planned home visits by CHW may be more feasible compared to a scientific and a state-of-the-art door-to-door approach, even at the risk of missing cases. The observed spill over-effect of unscreened PwE from within the catchment area, voluntarily presenting to the neurology consultation, may have offset missed persons during screening.

## Conclusion

This project supports the mhGAP recommendation to engage CHWs in mental and neurological health activities. Our data demonstrate the feasibility and effect of a short-term project on identification and mobilization of PwE when involving CHWs equipped with an easy-to-administer epilepsy screening tool. We recommend targeted scaling-up mobilization campaigns engaging CHWs, given the high prevalence of epilepsy in Rwanda, if adequate access to care and treatment is guaranteed.

## Supplementary Information

**Additional file 1.** Limoges epilepsy screening questionnaire in English and Kinyarwanda.

## Data Availability

The data supporting the findings of the study can be obtained on request from Dr. Peter Dedeken, corresponding author. Request will be assessed for data release by the team within a reasonable timeframe.
