# Supplementary material for: Effectiveness of community health workers on identification and mobilization of persons living with epilepsy in rural Rwanda using a validated screening tool
Source: Hum Resour Health. 2022 Jan 21;20:10. doi: 10.1186/s12960-022-00704-5 (PMC8780363; doi:10.1186/s12960-022-00704-5)
Supplement: Supplementary file 1 — Additional file 1. Limoges epilepsy screening questionnaire in English and Kinyarwanda. [file 12960_2022_704_MOESM1_ESM.docx]

| **Question** | **English** | **Kinyarwanda** |
| --- | --- | --- |
| 1 | Have you experienced a loss of consciousness and/or loss of urine and /or presence of foam? | Guta ubwenge cg kwinyarira cg guta urukonda? |
| 2 | Have you experienced a brief absence and/or loss of contact with those around you, of abrupt onset and of short duration? | Guta ubwenge cg guhungetwa bitunguranye kandi bikamara akanya gato? |
| 3 | Have you presented uncontrollable jerks or abnormal movements in one or more limbs (convulsions), of sudden onset and lasting a few minutes? | Kugagara k'urugingo cg ingingo bitangira rimwe kuri rimwe kandi bikamara igihe gito? |
| 4 | Have you experienced a sudden and brief onset of strange bodily sensations, hallucinations or visual, auditory and olfactory illusions? | Kumva umubiri uhindutse ku buryo budasanzwe, kubona, kumva cg guhumurirwa n'ibintu abandi batabona bitangira rimwe kuri rimwe kandi bikamara akanya gato? |
| 5 | Have you been told you had epilepsy or that you presented epilepsy in the past? | Bigeze bakubwira ko waba urwara igicuri cyagwa waba waragize ibimenyetso bya cyo? |

**Additional file 1** Limoges epilepsy screening questionnaire in English and Kinyarwanda (^1^)

**Reference:**

1 Preux P-M, Druet-Cabanac M, Debrock C, Philippe T, Dumas M: Questionnaire d’investigations de l’épilepsie dans les pays tropicaux. [Questionnaire in a study of epilepsy in tropical countries]. *Bull Soc Pathol Exot* 2000; 93:276-278 and suppl 93:1-22.
